# The effects of intestinal microbial community structure on disease manifestation in IL-10^-/-^ mice infected with *Helicobacter hepaticus*

**DOI:** 10.1186/2049-2618-1-15

**Published:** 2013-05-10

**Authors:** Nabeetha A Nagalingam, Courtney J Robinson, Ingrid L Bergin, Kathryn A Eaton, Gary B Huffnagle, Vincent B Young

**Affiliations:** 1 Department of Microbiology and Molecular Genetics, Michigan State University, East Lansing, MI, 48109, USA; 2 Department of Internal Medicine/Infectious Diseases Division, University of Michigan Medical School, Ann Arbor, MI, 48109, USA; 3 Unit for Laboratory Animal Medicine and Department of Microbiology and Immunology, University of Michigan Medical School, Ann Arbor, MI, 48109, USA; 4 Department of Internal Medicine/Pulmonary Division, University of Michigan Medical School, Ann Arbor, MI, 48109, USA; 5 Department of Microbiology and Immunology, University of Michigan, Ann Arbor, MI, 48109, USA; 6 Current address: Department of Medicine, Division of Gastroenterology, University of California San Francisco, San Francisco, CA, 94143, USA; 7 Current address: Department of Biology, Howard University, Washington, DC, 20059, USA

**Keywords:** Microbiota, IBD, *Helicobacter hepaticus*, IL-10^-/-^ mice, 16S rRNA

## Abstract

**Background:**

The aberrant inflammation that is the hallmark of the inflammatory bowel diseases (IBD) is associated with several factors, including changes in the intestinal microbiota. Here, we confirmed that an intestinal microbiota is needed for development of typhlocolitis in *Helicobacter hepaticus* infected IL-10^-/-^ C57BL/6 mice, and investigated the role of the microbiota in modulating disease.

**Results:**

We altered the murine microbiota by treatment with the antibiotics vancomycin or cefoperazone prior to *H. hepaticus* infection. Through surveys of the 16S rRNA encoding-gene, analyses of histology and changes in expression of host mediators, we correlated alterations in the microbiota with host responses. We found that resident microbes are essential for initiation of disease, as animals mono-associated with *H. hepaticus* did not develop colitis. Despite the requirement for an indigenous microbiota for the initiation of disease, the severity of disease was independent of antibiotic-induced changes in the microbial community structure. Despite differences in the expression of host inflammatory mediators associated with shifts in the microbiota, *H. hepaticus* infection led to similar histopathologic lesions in microbial communities exposed to either cefoperazone or vancomycin.

**Conclusion:**

In conclusion, we demonstrate that colitis due to *H. hepaticus* infection can be initiated and progress in the presence of several different microbial communities. Furthermore, *H. hepaticus* is the main driver of inflammation in this model, while the specific structure of the microbiota may modulate the host pathways that lead to chronic inflammation.

## Background

Inflammatory bowel diseases (IBD), of which Crohn’s disease (CD) and ulcerative colitis (UC) are the most common, are a group of idiopathic conditions that result in inflammation of the intestinal mucosa. Although the incidence of IBD is increasing, its cause is yet to be determined [1,2]. However, recent evidence indicates that genetic factors, dysregulated immune responses and altered intestinal microbial communities play key roles in the onset of disease [3].

Alteration of microbial community structure has been associated with intestinal diseases in patients and animal models of IBD [4-8]. For example, fecal and biopsy samples from IBD patients have different microbial communities to those from healthy controls [9]. Furthermore, patients with CD contain communities that are distinct from those in UC [9]. Animal models have been key, not only in elucidating the importance of interactions between the immune system and microbiota, but also the importance of microbial community structures in IBD. Knock-out mice that are double-deficient in both *Tbet*, a transcriptional factor, and *Rag2*, recombination activating gene, contain colitogenic communities that can be transferred into healthy recipients who subsequently develop disease [10]. Additionally, inflammation does not develop in germfree mice in some models, providing further evidence for the necessity of resident communities, and the significance of microbial community structure, in the development of enteric diseases [11-13]. Results of these studies suggest that changes in the balance among members of the indigenous microbial community can trigger an inflammatory response.

Multiple animal models have been developed to study IBD and, more recently, several of these have been used to monitor how changes in microbial communities affect disease [14,15]. We have previously employed a specific murine model that involves infection of IL-10-deficient mice (IL10^-/-^ C57BL/6) with *Helicobacter hepaticus*. In this model, *H. hepaticus* infection elicits a dysregulated Th (T helper)1/Th17 response that results in clinical and histopathologic disease that resembles Crohn’s disease in humans [16,17]. Infection with *H. hepaticus* does not cause disease in wild-type mice and only triggers inflammation in immune-altered hosts [18,19]. Onset of disease is MyD88-dependent [20].

Interestingly, *H. hepaticus* only causes disease in the presence of resident microbiota as there is no disease observed in ex-germfree mice mono-associated with *H. hepaticus*[21], indicating the importance of the microbiota in initiation of IBD. There is also evidence that mouse strain influences *H. hepaticus*-induced alterations in the microbiota that may be related to disease manifestation [22]. However, apart from these findings, there is limited knowledge of the roles that the resident microbiota play in this model. Therefore, the aim of the current study was to investigate roles of the microbiota in *H. hepaticus*-triggered IBD by observing how altering community structure of the indigenous microbiota can alter disease manifestations. Previously, we showed that treatment with antibiotics, cefoperazone and vancomycin significantly alter microbial communities of the murine gut [23,24]. In the current study, we used these antibiotics to modify the community structure in the guts of IL10^-/-^ C57BL/6 mice before and during infection with *H. hepaticus*, and then compared disease in treated and untreated mice as well as germfree mice. Cefoperazone has not been cited to have activity against *H. hepaticus*, but this bacterium is resistant to vancomycin [25]. Our goal was to determine whether presence or absence of microbiota or alterations in microbiota structure would alter disease severity in IL10^-/-^ C57BL/6 mice infected with *H. hepaticus*.

## Methods

### Animals

IL-10^-/-^ mice on a C57BL/6 background, from a breeding colony (Michigan State University and the University of Michigan) initially established with breeding stock from Jackson Laboratories (Bar Harbor, ME, USA), were used for experiments. Mice were housed with autoclaved bedding, given sterile food and water *ad libitum,* and exposed to 12:12 h light:dark cycles. Germfree IL10^-/-^ C57BL/6 mice were from the University of Michigan Germ Free Life Laboratory. They were maintained in sterile bubble isolators, and they remained bacteriologically sterile until removed from the isolators and inoculated with *H. hepaticus*. All animal studies were conducted at the University of Michigan and were approved by the University Committee on Use and Care of Animals (UCUCA).

### Murine infection with *H. hepaticus*

As previously described, the wild-type *H. hepaticus* strain 3B1 (ATCC 51449) was incubated at 37°C under micro-aerobic conditions for 48 h, on tryptic soy agar (TSA) plates, supplemented with 5% sheep’s blood, and then re-suspended in 5 ml of tryptic soy broth (TSB) [19]. Mice were inoculated with a suspension of bacteria with an optical density of 1.0 at 600 nm (approximately 10^8^ colony-forming units (CFU)) in a volume of 0.2 ml. Bacteria were introduced directly into the gastrointestinal tract with a 24-gauge ball-tipped gavage needle. Control mice were inoculated with sterile TSB [19].

**Figure 1:**
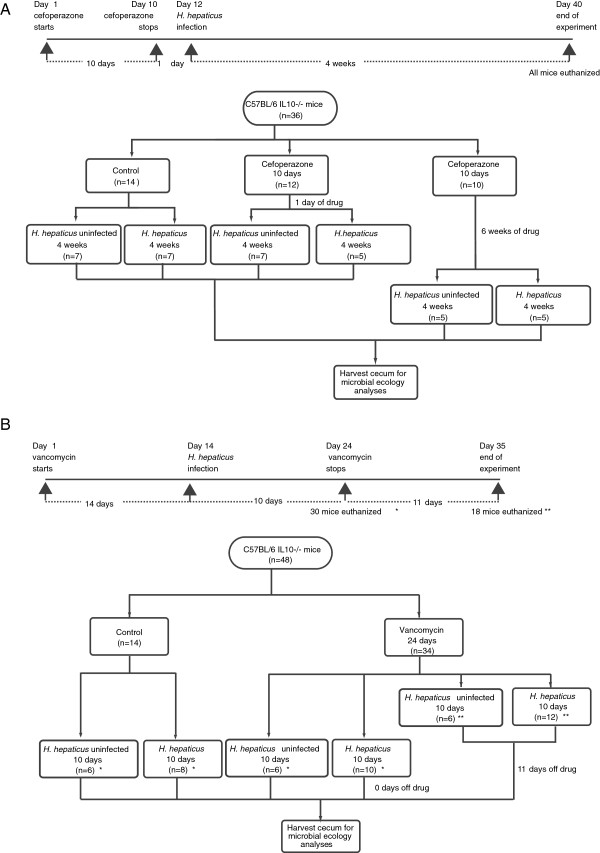
**Schematic of experimental designs showing cefoperazone and vancomycin administration to IL10 **^**-/- **^**C57BL/6 mice prior to or during infection with *H. hepaticus*.** (**A**) Cefoperazone administration. (**B**) Vancomycin administration. Scales represent the Morisita-Horn index describing dissimilarity distance among communities.

Three experimental studies were conducted to investigate roles of the microbiota in disease development. The first was to investigate disease development in *H. hepaticus* mono-associated IL10^-/-^ C57BL/6 mice from our colonies, two groups of germfree mice were used: five mice were gavaged with TSB only and five were gavaged with the *H. hepaticus* suspension described above. Tissues were collected four weeks post-infection. The second and third experiments were to investigate the roles of altered microbiota in disease development. We manipulated microbial communities using two different antibiotic treatments, cefoperazone and vancomycin. Cefoperazone was administered, *ad libitum*, as a 0.5 mg/ml solution in the drinking water of 10-week-old, conventionally raised IL10^-/-^ C57BL/6 mice. Mice were assigned to six groups, as shown in Figure 1A. Vancomycin treatment involved *ad libitum* administration of 0.1 mg/ml vancomycin in the drinking water of 6- to 8-week-old, conventionally raised IL10^-/-^ C57BL/6 mice [24]. These mice were assigned to six experimental groups as depicted in Figure 1B.

### Necropsy and histology

Mice were euthanized by CO_2_ asphyxiation and cecal samples were collected as follows [26]. Cecal tips were excised and opened along the longitudinal axis. Contents were removed, and cecal walls were gently washed with 1 × PBS to remove luminal contents. Tissues were cut into three 3 × 3 mm sections and snap-frozen in liquid nitrogen before storing at -80°C until future use. The remaining portions of cecum were processed for histology by removing luminal contents before washing with PBS, and placing in tissue cassettes. Cassettes were then submerged in 10% neutral buffered formalin for 24 h and transferred to a 70% ethanol solution prior to processing for paraffin embedding and sectioning at 5 microns, and staining with H & E.

Stained slides were blinded and scored. The following scoring system was used: 0, no significant inflammation; 1, small multifocal lamina proprial or transepithelial leukocyte aggregates; 2, coalescing lamina proprial leukocyte aggregates, may have submucosal involvement; 3, frequent coalescing leukocyte aggregates with submucosal involvement, may have follicle formation; 3.5, strong submucosal component, infrequent extension to muscularis externa or mesocolon; 4, diffuse or regionally extensive transmural involvement.

### DNA extraction

Genomic DNA was extracted from tissue using the MagNA Pure® system and the Nucleic Acid Extraction Kit I (Roche, Indianapolis, IN, USA) according to the manufacturer’s directions. Briefly, tissue samples were incubated in lysis buffer and proteinase K at 56°C, before being placed in the MagNA Pure® Compact machine for purification of DNA. Resulting DNA was then used in preparation of clone libraries and quantitative PCR (qPCR).

### Clone library construction

**Figure 2:**
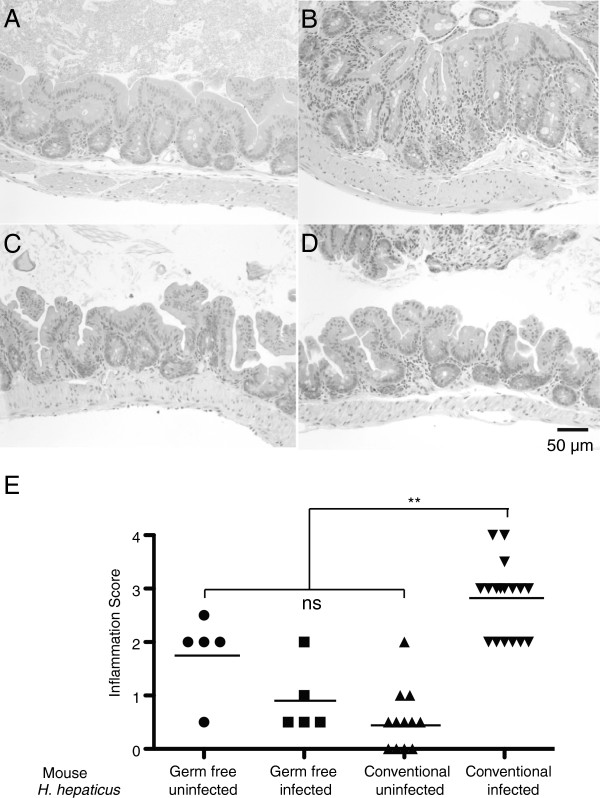
***H. hepaticus *triggered inflammation in conventionally raised IL10**^**-/- **^**C57BL/6 mice compared to germfree counterparts.** Representative histologic sections of mouse cecum are depicted. (**A**) *H. hepaticus-*uninfected conventionally raised mice appear normal. (**B**) *H. hepaticus*-infected conventional section shows edema, hyperplasia and inflammatory infiltrate. (**C**) *H. hepaticus-*uninfected germfree control, and (**D**) *H. hepaticus-*infected germfree cecum sections are both normal, with few signs of inflammation. (**E**) Quantification of indicators of pathology showing significant inflammation only in histologic sections from *H. hepaticus-*infected conventionally raised mice. *Statistically significant changes (*P* <0.05).

PCR targeting the 16S rRNA-encoding gene was performed as previously described in Young *et al.*[27]. PCR reactions were prepared using broad-range primers, (8 F, 5'-AGAGTTTGATCCTGGCTCAG-3'; 1492R, 5'-GGTTACCTTGTTACGACTT-3'). Approximately 100 ng/μl of DNA extracted from cecal tissue samples were added to each PCR reaction. Each 25 μl PCR mixture contained 20 pmol of each primer, 200 μM of each deoxynucleoside triphosphate, and 1.5 U of Taq DNA polymerase in a final concentration of 10 mM Tris–HCl, 50 mM KCl, and 1.5 mM MgCl_2_ (illustra PuReTaq™ Ready To Go PCR™ beads; Amersham Pharmacia Biotech, Piscataway, NJ, USA). Cycling conditions included 20 cycles of 30 seconds at 94°C, 45 seconds at 54°C, and 45 seconds at 72°C. PCR products were visualized by agarose gel electrophoresis. GE illustra™ Microspin™ S-400 HR columns were used to purify amplicons (GFX, GE Healthcare, Piscataway, NJ, USA) as directed by the manufacturer. Purified PCR products were ligated into the TOPO 4® vector (Invitrogen K4575-01, Carlsbad, CA, USA) according to the manufacturer’s specifications, and transformed into *Escherichia coli* TOP10 cells. Clones were grown overnight at 37°C in Luria Broth (LB) amended with carbenicillin (50 μg/ml)). A total of 3,958 clones were sequenced, with an average of 329 clones per treatment group (detailed in Figure 2).

Vector-specific primers (M13F, 5'-CAGTCACGACGTTGTAAAACGACGGC-3'; and M13R, 5'-CAGGAAACAGCTATGACCATG-3') were used to screen for clones containing the appropriately sized inserts and to amplify inserts for sequencing. Partial 16S rRNA gene sequences were generated using primer 8 F, at the Sequencing Core at the University of Michigan. Raw sequence data were processed through an automated information pipeline available through the Ribosomal Database Project (RDP) website (http://rdp.cme.msu.edu/). Distance matrices containing data from each library were calculated from alignments generated by the RDP aligner [28]. The program, mothur [29], was used to assign sequences to operational taxonomic units (OTUs) based on values in the distance matrices. Analyses were done using a 95% sequence similarity to denote genus level [29], and 97% for species level. EstimateS [30] and mothur were used to calculate ecological measures from the distance matrices. The inverse Simpson diversity index was used to indicate diversity, a measure of richness and evenness. The Morisita-Horn index was used to measure structural similarity among samples, taking into account both abundance and membership of the community. Dendrograms were generated using the Mega3 program, and the parsimony test from the mothur suite of programs was used to denote significant differences between treatment groups [29,31]. Additionally, taxonomic assignments were determined using Classifier through the RDP website (80% confidence cutoff).

### qPCR analysis of microbial communities

The quantity of 16S rRNA operons in the samples relative to a single-copy host gene was measured using a primer/probe set that targets a broad range of rRNA-encoding gene sequences (forward primer (5′-TCCTACGGGAGGCAGCAGT-3′); reverse primer (5′- GGACTACCAGGGTATCTAATCCTGTT-3′); and probe (5′-(6-FAM)- CGTATTACCGCGGCTGCTGGCAC-(TAMRA)-3′)) [32]. A primer/probe set targeting a 264-bp portion of the TNF alpha gene was used as a reference using 200 nanomoles of the forward (TNFα_mu_se; 5′-GGCTTTCCGAATTCACTGGAG-3′) and reverse primers (TNFα_mu_as; 5′- CCCCGGCCTTCCAAATAAA-3′), and 100 nanomoles of the probe (TNFα_mu_probe; 5′-(Cy5)-ATGTCCATTCCTGAGTTCTGCAAAGGGA-(Iowa Black RQ™)-3′) [33]. The reaction mix consisted of LightCycler® 480 Probes Master reaction mix (Roche) at 1 × concentration, and appropriate primer/probe pair. Amplification of each gene was done under separate run conditions: the 16S rRNA gene target had an activation step of 50°C for 2 minutes followed by 95°C for 10 minutes. Forty-five cycles were done at 95°C for 15 s and 60°C for 1 minute before holding at 4°C. The TNF reference gene was also amplified with an activation step of 50°C for 2 minutes followed by 95°C for 10 minutes. Forty-fives cycles of 95°C for 20 s and 64°C for 30 s were done before holding at 4°C. Calculations of 2-ΔΔ cycle threshold (Ct) were made to compare changes in the amount of 16S rRNA operons from samples between treatment groups.

*H. hepaticus* was quantified as describes by Ge *et al*.[34]. Briefly, the *H. hepaticus cdtB* gene was amplified using a primer/probe set of 400 nM forward primer (5′-CCGCAAATTGCAGCAATACTT-3′), reverse primer (5′-CACCTGTGCATTTTGGACGA-3′), and 100 nM probe (5′-[FAM]-AATATACGCGCACACCTCTCATCTGACCAT-(TAMRA)-3′). The reaction mix consisted of LightCycler® 480 Probes Master reaction mix (Roche) at 1X concentration, and run conditions of 50°C for 2 min, 95°C for 10 min, and then 40 to 45 repeats of 95°C for 15 s and 60°C for 60 s. The TNF reference gene was also amplified, as described above, for 2^-ΔΔCt^ calculations.

### RNA extraction and determination for cytokine expression

Total nucleic acids were extracted using MagNA Pure® (Roche) and the RNA Extraction Kit as directed by the manufacturer’s protocol. Total RNA was then isolated from three mice per treatment group by treating total nucleic acids with RNase-free, recombinant DNaseI, (Roche, Indianapolis, IN, USA), and quality checked via PCR for DNA contamination. The High-Capacity cDNA Reverse Transcription Kit (Applied Biosystems, Foster City, CA, USA) was used to transcribe total RNA to cDNA. Cytokine expression was determined by applying the cDNA to a custom Superarray® (Frederick, MD, USA) PCR plate which contained gene-specific primers designed for the following targets [35]: IFNγ, IL-12a, TNFα, IL-4, IL-13, IL-5, TGFβ, IL-6, forkhead box (FOX)p3, IL-17a, IL-23a, CXCL2, CCL5, nitric oxide synthase (NOS)2, arginase (Arg)1, chitinase (Chi)3/4, prostaglandin-endoperoxide synthase (PTGS)2, indoleamine-pyrrole 2,3-dioxygenase (INDO), glyceraldehyde-3-phosphate dehydrogenase (GAPDH) (used in normalization calculations), a reverse-transcription control and a genomic DNA control. Quantification was done with LightCycler® 480 SYBR Green I Master and analyzed on a LightCycler® 480 system (Roche Diagnostics, Indianapolis, IN, USA) as directed by the manufacturer’s protocol, with the following program: 95°C activation for 10 minutes; 40 cycles of 95°C denaturation for 15 s, and 60°C annealing for 1 minute. Resulting threshold values were analyzed by calculating the 2-ΔΔCt values, using GAPDH as the reference, to calculate fold regulation compared to the antibiotic untreated, *H. hepaticus* uninfected control [36-38].

### Statistical analysis

Statistical significance in the histological scores were determined using the non-parametric Kruskal-Wallis test. Comparisons of specific microbial taxa was accomplished by Fisher’s exact test using Metastats, with a false discovery rate correction to provide a prioritized list of microbial abundances among the treatment groups [39]. Differences in clustering within dendrograms were determined using the parsimony test function in mothur [29]. Probability values less than 0.05 were considered significantly different.

## Results

### Germfree IL10^-/-^ C57BL/6 mice infected with *H. hepaticus* do not develop colitis

To confirm whether resident microbiota have an important role in the development of disease in IL10^-/-^ C57BL/6 mice, germfree animals were mono-associated with *H. hepaticus*. The relative levels of *H. hepaticus* were measured via qPCR and indicated that the organism burden in germfree mice was not significantly different from that encountered in conventional mice (-2.94 ± 1.90 fold change, *P* = 0.446) (Table 1).

**Table 1:** **Quantitative PCR measures of fold changes in the total bacterial and in *H. hepaticus *** **loads from genomic DNA extracted from cecum**

**Treatment**		**Fold change ± SD**	
	***H. hepaticus *** **infected**	**Total bacterial load**	***H. hepaticus *** **load**
Cefoperazone (cef) treatment			
Control 1 (no cef)	No	calibrator	N/A
Control 2 (1 day recovery from cef)	No	-3,333.00 ± 0.34	N/A
Control 3 (6 wk recovery from cef)	No	2.16 ± 1.18	N/A
Community 1 (no cef)	Yes	1.25 ± 0.75	calibrator
Community 2 (1 day recovery from cef before infection)	Yes	4.48 ± 2.55	-1.12 ± 2.79
Community 3 (6 wk recovery from cef before infection)	Yes	-1.35 ± 0.71	3.94 ± 6.30
Vancomycin (vanc) treatment			
Control 4 (no vanc)	No	1.17 ± 0.68	N/A
Control 5 (vanc)	No	1.95 ± 1.41	N/A
Control 6 (vanc + 11 days recovery)	No	1.41 ± 0.91	N/A
Community 4 (no vanc)	Yes	1.90 ± 1.91	1.04 ± 0.30
Community 5 (vanc)	Yes	1.98 ± 1.62	-1.59 ± 0.52
Community 6 (vanc + 11 days recovery)	Yes	1.14 ± 1.16	1.28 ± 0.56
Germfree mice infected with *H. hepaticus*		N/A	-2.94 ± 1.90

The fold change reported (2 -ΔΔCt) is normalized to TNFα and is in comparison to the samples indicated as calibrator in the respective columns. N/A, not applicable.

Significantly less inflammation was detected in ceca of germfree mice infected with *H. hepaticus* compared to infected conventional animals (Figure 2). This result is in agreement with a previous study [21]. Uninfected IL10^-/-^ C57BL/6 mice, from our breeding colony, raised in specific pathogen-free conditions did not develop inflammation that was significantly different from conventionally raised wild-type animals.

### Cefoperazone alters total bacterial load, but not *H. hepaticus*-induced disease severity

To determine the effect of microbiome population size and composition on experimental colitis, we altered the community by reducing bacterial loads (via cefoperazone), prior to *H. hepaticus* infection. This treatment resulted in a more than 1,000-fold decrease in bacterial load one day after ending cefoperazone administration (Table 1), without altering the community structure. After cefoperazone treatment, mice were administered untreated water and allowed to recover for one day*,* or for six weeks before infecting with *H. hepaticus*. Four weeks post infection, all communities had similar total bacterial loads compared to control animals that did not receive cefoperazone nor were infected with *H. hepaticus* (*P* = 0.224), indicating that the communities had recovered during the four weeks. Communities in infected mice contained similar amounts of *H. hepaticus* regardless of cefoperazone treatment or recovery period (*P* = 0.246) (Table 1). It is noteworthy that there was an exception, with one infected mouse having 20-fold lower detectable *H. hepaticus*.

**Figure 3:**
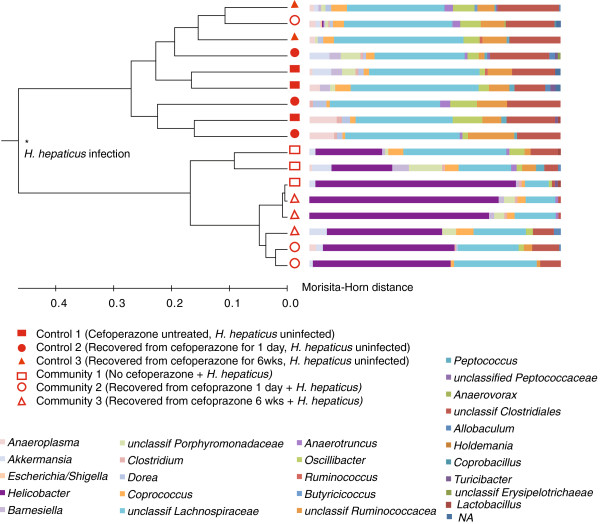
***H. hepaticus *infection significantly alters the community structure of mice even four weeks after termination of cefoperazone treatment.** *Statistically significantly different community structures calculated by the parsimony test (*P* <0.05).

Clone libraries were constructed from three individual mice from each treatment group (Table 2). OTU-based analyses indicate that the communities in *H. hepaticus*-infected mice were significantly different from those in mice that did not harbor *H. hepaticus* (*P* <0.001). This difference was due to the presence of *H. hepaticus*, as no statistically significant differences were found among communities when *H. hepaticus* sequences were masked. In communities of infected mice, the relative abundance of *H. hepaticus* varied between 25 and 70% (Figure 3). This difference in relative abundance of *H. hepaticus* was independent of cefoperazone treatment. The outlier mouse that contained low numbers of *H. hepaticus,* as determined via qPCR, contained a community that produced only two of ninety clones that affiliated with *Helicobacter.* This supports the finding that *H. hepaticus* did not dominate this community as was typically seen in the other mice in this treatment group.

**Table 2:** Clones generated from genomic DNA extracted from cecum samples

**Treatment**		
	***H. hepaticus *** **infected**	**Clones/sample (number)**
Cefoperazone (cef) treatment		
Control 1 (no cef)	No	93, 54, 93 (3)
Control 2 (1 day recovery from cef)	No	90, 75, 92 (3)
Control 3 (6 wk recovery from cef)	No	95, 90 (2)
Community 1 (no cef)	Yes	83, 92, 86 (3)
Community 2 (1 day recovery from cef before infection)	Yes	150, 163, 76 (3)
Community 3 (6 wk recovery from cef before infection)	Yes	93, 72, 58 (3)
Vancomycin (vanc) treatment		
Control 4 (no vanc)	No	95, 95, 95 (3)
Control 5 (vanc)	No	94, 84, 94 (3)
Control 6 (vanc + 11 days recovery)	No	95, 93, 92 (3)
Community 4 (no vanc)	Yes	89, 89, 92, 93, 94 (5)
Community 5 (vanc)	Yes	94, 95, 93, 90, 92, 94 (6)
Community 6 (vanc + 11 days recovery)	Yes	94, 91, 92, 93, 91, 90, 96 (7)

There were reductions in the genus *Clostridia* (*P* = 0.032) and family *Lachnospiraceae* (*P* = 0.016) in communities infected with *H. hepaticus* after only one day off cefoperazone compared to those infected after six weeks of recovery. This indicates differences in recovery dynamics in the two treatment groups.

**Figure 4:**
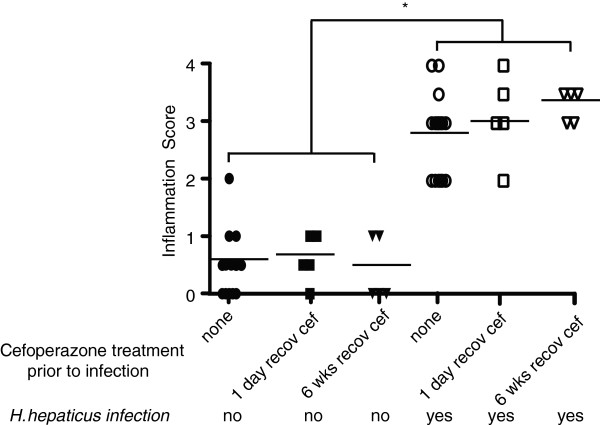
**Blinded scores showing significant inflammation.** Compared to uninfected controls, in all *H. hepaticus-*infected cecal sections regardless of cefoperazone treatment. *Statistically significant changes (*P* <0.05).

To investigate whether initially low bacterial loads would affect *H. hepaticus-*induced disease in IL10^-/-^ C57BL/6 mice, cefoperazone was administered to mice before infecting with the bacterium. Cecal sections were scored, and revealed that all animals infected with *H. hepaticus* developed inflammation (Figure 4). There was no correlation between disease scores and relative abundance of *H. hepaticus* (Spearman’s rank correlation coefficient *P* = 0.242). Despite reduction in initial microbial loads of cefoperazone-treated communities, disease severity was unaffected in *H. hepaticus-*infected mice as compared to infected mice that were not administered antibiotics.

### Vancomycin alters community structures, but not *H. hepaticus*-induced disease severity

**Figure 5:**
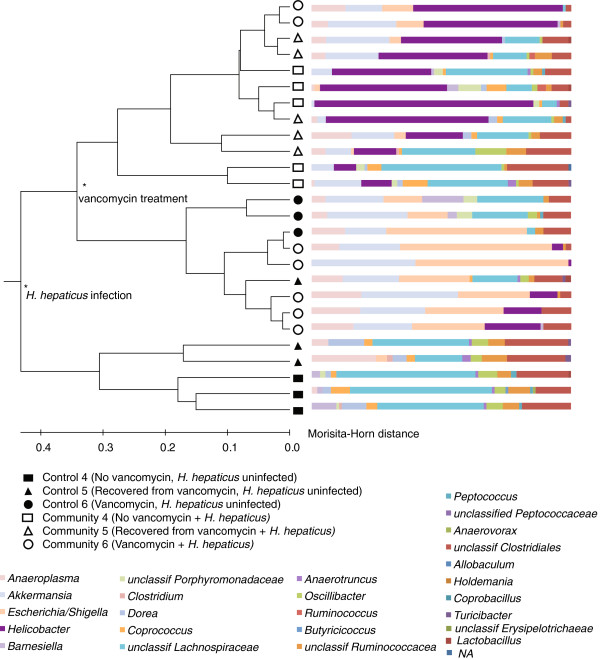
***H. hepaticus *infection, as well as vancomycin treatment, significantly alters the intestinal community structure.** *Statistically significantly different community structures calculated by the parsimony test (*P* <0.05).

In order to investigate the effects of altering community structure on the development of inflammation, vancomycin was administered to mice prior to *H. hepaticus* infection. Through clone library construction, we found that vancomycin-treated communities were significantly altered and these changes persisted even after antibiotic administration ceased. Although bacterial load measured by qPCR, targeting the 16S rRNA-encoding gene, was not altered by vancomycin treatment (Table 1), this antibiotic caused a decrease in overall microbial diversity (an inverse Simpson’s index value of 6.57 (± 1.92) compared to 34.6 (± 8.22) for control communities) and a slight, but not significant, increase in relative abundance of members of the Proteobacteria, namely *E.coli/Shigella* (not detected in any control communities, detected in all vancomycin communities at 14.9 to 54.8%, *P* = 0.06; Figure 5). These results indicate that vancomycin alone causes changes in the microbial community.

Post-infection studies revealed that mice that received vancomycin both before and after infection with *H. hepaticus* contained a slightly reduced, but not significantly different burden of *H. hepaticus* compared to mice that did not receive antibiotics (*P* = 0.06). Additionally, mice that received vancomycin before and after infection contained fewer *H. hepaticus* than mice that had been administered vancomycin previous to *H. hepaticus* infection, but not after (*P* = 0.009). There was no difference between the *H. hepaticus* loads in mice that were infected but did not receive vancomycin and mice that were treated with vancomycin only prior to infection (*P* = 0.28).

Structurally, the microbial communities in mice that were treated with vancomycin prior to infection were more similar to communities in mice that were infected without receiving antibiotics than those in mice that received vancomycin before and after infection (Figure 5). Compared to the communities of infected mice that were not exposed to vancomycin, infected mice that were administered vancomycin continuously contained communities with decreased population sizes of *Oscillibacter* (*P* = 0.028), *Coprococcus* (*P* = 0.008), *Dorea* (*P* = 0.029) and unclassified members of the families Lachnospiraceae (*P* = 0.022), and Porphyromonadaceae (*P* = 0.008). Additionally, the communities in infected mice that were continuously administered vancomycin exhibited increases in *E. coli/Shigella* (*P* = 0.001), *Akkermansia* (*P* = 0.001) and *Anaeroplasma* (*P* = 0.001). Taxonomic analyses also indicated that communities in mice that received vancomycin only before infection exhibited a significant increase in *Coprococcus* (*P* = 0.006) and *Dorea* (*P* = 0.011), as well as unclassified members of the Lachnospiraceae (*P* <0.001) and Ruminococcaceae (*P* = 0.020), compared to communities in infected mice treated with vancomycin continuously. Furthermore, the communities that were exposed to vancomycin prior to infection contained decreased populations of *E. coli/Shigella* (*P* = 0.001) compared to communities in mice that were infected and maintained on vancomycin treatment (Figure 5).

**Figure 6:**
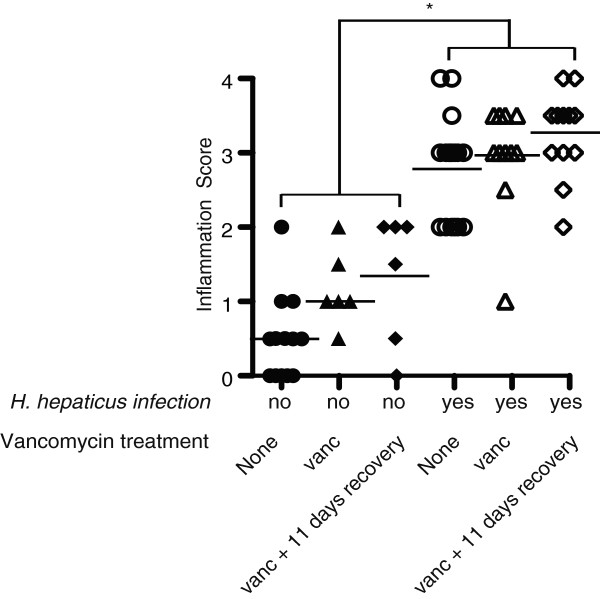
**Blinded scores showing that inflammation in all *H. hepaticus-*infected mice were greater than in uninfected mice, regardless of vancomycin treatment.** *Statistically significant changes (*P* <0.05).

To investigate the effects of vancomycin-induced changes to the microbiota on disease manifestation, cecal sections of vancomycin-treated mice were examined and scored for inflammation and hyperplasia. All animals infected with *H. hepaticus* developed similar levels of histopathologic disease, regardless of the shifts in the microbial community structures (Figure 6).

### Infection with *H. hepaticus* and antibiotic administration induce host responses

Although severity of inflammation was not altered by changes in initial bacterial load (via cefoperazone treatment) or shifts in resident community structure (via vancomycin treatment), we questioned whether differential immunologic changes were occurring within the host at a finer resolution. To investigate possible host effects, we measured changes in expression of host mediators in animals that were treated with antibiotics prior to *H. hepaticus* infection and compared this to the host responses in animals that did not receive antibiotics prior to *H. hepaticus* challenge. As a control, we also measured the host responses in animals that received the antibiotic treatments but were never challenged with *H. hepaticus*.

**Figure 7:**
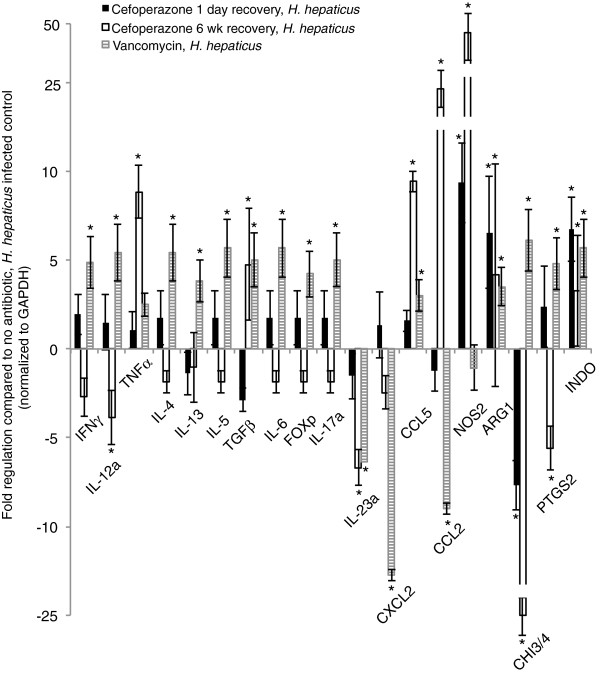
**Changes in the expression of host mediators in cecal tissue of mice administered antibiotics and infected with *H. hepaticus*.** Cefoperazone with one-day recovery, black bars; cefoperazone with 6 weeks recovery, white bars; vancomycin, hatched bars; *P* <0.05. *Host mediators that are significantly upregulated, or downregulated compared to expression of host mediators from mice infected with *H. hepaticus,* and not administered antibiotics.

In mice that were challenged with *H. hepaticus* following cefoperazone treatment, there was a significant upregulation of several host mediators including TNFα, CCL5, CCL2, Nos2 and Arg1, or downregulation in Chi3/4 and IL-23a (Figure 7). Even when mice were allowed to recover for 6 weeks after cefoperazone treatment ended, expression of some mediators remained significantly different from *H. hepaticus-*infected mice that did not receive cefoperazone, indicating that treatment with the antibiotic is associated with an altered immune response in the presence of this bacterium, although these changes did not affect the degree of histopathologic disease. In addition, vancomycin administration resulted in significant upregulation of several host mediators including INFγ, IL-12a, IL-17a and Chi3/4; and significant downregulation of other genes such as CXCL2 and CCL2 (Figure 7). The changes following vancomycin were not completely overlapping with those seen in cefoperazone-treated mice and in some cases were opposite those seen in cefoperazone-treated animals. These findings indicate that antibiotic treatment can have long-term effects on host response, and different host responses are elicited by different antibiotic-induced changes, in communities containing *H. hepaticus*. In animals that received antibiotics alone, without subsequent *H. hepaticus* infection, cefoperazone induced changes in the expression of several host response genes (Table 3), but these changes alone did not explain the findings in infected mice. Only the expression of Ccl2 was minimally increased in vancomycin-treated animals. Larger numbers of changes were seen in cefoperazone-treated animals, and the greatest changes were seen in animals that only recovered from cefoperazone for one day.

**Table 3:** **Changes of cytokine gene expression in antibiotic treated, *Helicobacter hepaticus *** **uninfected mice compared to untreated, uninfected controls**

	**Vancomycin**		**One-day recovery from cefoperazone**		**Six-week recovery from cefoperazone**	
**Cytokines**	***H. hepaticus *** **uninfected**	**SD**	***H. hepaticus *** **uninfected**	**SD**	***H. hepaticus *** **uninfected**	**SD**
INFg	1.13	0.66	1.64	1.31	-3.78	1.10*
IL12a	1.13	0.83	8.88	0.64*	-3.58	1.10*
TNF	1.28	0.21	1.56	0.27	1.64	6.12
IL4	1.13	0.58	1.96	1.07	-1.6	1.1
IL13	1.60	0.21	9.25	0.83*	1.51	1.1
IL5	1.13	0.58	-21.48	1.36*	-1.6	1.1
TGF	-1.05	0.59	-51.09	1.36*	-3.29	1.40*
IL6	1.13	0.58	-21.48	1.36*	-1.6	1.1
FOXp3	1.13	0.58	-21.48	1.36*	-1.6	1.1
Il17a	1.68	1.16	-4.98	1.28*	-1.72	1.1
IL23	-1.05	0.48	4.92	0.62*	-1.6	1.1
Cxcl2	2.26	1.15	-5.76	2.35*	-1.6	1.1
Ccl5	-1.01	0.66	-4.07	0.54*	-5.84	7.73*
Ccl2	3.15	0.87*	9.61	0.35*	-10.2	1.10*
NOS2	2.94	1.30	-2.23	0.03	-3.93	3.34*
ARG1	1.13	0.58	6.41	0*	-1.62	0.81
Chi3/4	1.13	1.19	16.45	0.26*	-13.69	1.10*
PTGS2	-1.05	0.93	1.22	0.01	2.46	4.95
INDO	1.60	0.81	-26.17	0.52*	-2.95	1.1

Results are presented as mean change. *Statistically significant difference. INF, interferon; IL, interleukin; TNF, tumor necrosis factor; TGF, transforming growth factor; Foxp3, Forkhead box protein 3; NOS, nitric oxide synthase; Chi, chitinase; PTGS, prostaglandin-endoperoxide synthase; INDO, indoleamine-pyrrole 2,3-dioxygenase.

## Discussion

Intestinal microbes have been credited with many beneficial functions as demonstrated by deficiencies in host development in animals raised in germfree conditions [40-43]. Collapse of the delicate balance between host and microbiota has been implicated in the onset of diseases such as IBD, a condition characterized by inappropriate host immune responses. IBD is also associated with an altered microbiota, often exhibited by patients as a reduction in diversity of the intestinal microbial community compared to healthy participants [44-47]. Additionally, some investigators noted a relative increase in particular bacterial groups such as Bacteriodetes in patients with IBD compared to healthy counterparts [48,49]. Such investigative studies have been important in characterizing the microbial community in the presence of disease, however, findings are difficult to interpret since the extent of the microbial community changes induced by host immune response is unknown. In our study, the microbial communities are altered before the induction of disease, thereby allowing us to ascertain the effects of community changes on disease development. There is also evidence that the microbial community is important in persistence of bowel inflammation, indicated by antibiotic studies showing amelioration of inflammation after treatment [50,51]. Apart from this, we have little insight into the role of the microbiota in IBD. We therefore used a murine model of IBD, *H. Hepaticus-*infected IL10^-/-^ C57BL/6 mice, to further investigate how changes in the microbial community are associated with disease. We accomplished this by testing whether the severity of disease is affected by different microbial community structures.

*H. hepaticus* is sometimes found as part of the resident intestinal communities of mice [52-54], and can induce disease in some cases. *H. hepaticus* can trigger inflammation in immuno-compromised mice such as Rag^-/-^, IL-10^-/-^ and SCID [22,55-57]. Furthermore, *H. hepaticus*-induced inflammation can be ameliorated by the use of antibiotics [58,59]. Also, *Helicobacter* causes disease in IL10^-/-^ C57BL/6 mice only in the presence of resident microbes, as seen here and by others [21], indicating an essential role of the indigenous community towards inducing inflammation in this model. We also found that *H. hepaticus* colonizes germfree mice with a similar burden as seen in conventionally raised animals. This indicates that *H. hepaticus* may have a particular niche that is unaffected by the presence of resident microbes.

Although other groups have shown the development of spontaneous disease in IL10^-/-^ mice in the absence of *H. hepaticus*[11,21], we observed that less than 5% of mice in our breeding colony developed disease over a 2-year period [19], suggesting that disease can be associated with several microbial structures. These data collectively suggest that certain microbiota are important drivers of inflammation in IBD, particularly in this model, making it an appropriate system in which to study the effects of an altered microbial community structure on severity of inflammation.

Others have shown that when mice were infected with *H. hepaticus,* and were mono-associated with *Lactobacillus reuteri*, they developed more inflammation than *H. hepaticus* uninfected animals, emphasizing the need for microbial interaction in disease onset [60]. To investigate the extent of the role of the microbiota, we infected conventional IL10^-/-^ C57BL/6 mice, and mice treated with vancomycin and cefoperazone with *H. hepaticus* and assessed whether there were differences in disease severity. Two antibiotics, vancomycin and cefoperazone, were used to alter the microbial communities so that the effects of different community structures could be assessed in this genetically susceptible host. Each drug causes different changes in the gut community: vancomycin, a drug that targets the Gram-positive bacteria, caused shifts in the microbial structure without reducing the bacterial load, while cefoperazone, a broad-spectrum antibiotic, reduced the total bacterial load by over 1,000-fold, as we have shown here and previously [23,24]. With vancomycin, these shifts were mainly due to the reduction in Firmicutes, a phylum that typically comprises more than 50% of human gut community [27,61]. After a recovery period, communities exposed to vancomycin began to resemble control communities, but also exhibited lasting effects with a reduction in Firmicutes and an increase in Proteobacteria. Increases in Proteobacteria after vancomycin treatment have also been observed by other investigators [62], and is perhaps due to the reduction in Gram-positive bacteria, thereby making formerly occupied niches available to members of the phylum Proteobacteria. Previously, using 454 pyrosequencing analysis of the microbiota, we showed that Proteobacteria inhabit the ceca of IL10^-/-^ C57BL/6 mice [23]. It is likely that they were present in communities analyzed in the current study, but were below the limits of detection in clone libraries, a shortcoming we recognize in the Sanger sequencing used in this study compared to pyrosequencing techniques. Like other communities after a recovery period [27,63], cefoperazone-treated communities began to resemble communities in untreated controls, demonstrating resilience, even after a significant reduction in bacterial loads immediately after treatment.

In non-germfree mice, disease occurrence was independent of community structure with the only common significant association between disease and community change being the presence of *H. hepaticus*. Even then, the relative abundance of this bacterium did not correlate with disease score. This finding also resonates with the outcome that severe disease occurs even at very low colonization loads of *H. hepaticus*, just as it does with relatively high colonization levels. The most significant finding of this work is that the microbiota are essential for development of inflammation, and markedly different communities can facilitate development of IBD. This finding suggests that several non-*H. hepaticus* microbial members are involved in disease development, and *H. hepaticus* is the major driver of inflammation in this model.

The host immune response to *H. hepaticus*, such as increase in arginase and NOS2, observed in mice administered cefoperazone, was consistent with work by others which revealed increases in iNOS [64]. These enzymes are important in wound healing. Alterations in the microbial community by antibiotic treatment have been associated with altered immune responses [65,66], and since cefoperazone treatment is known to significantly reduce bacterial load in the intestine, we decided to further investigate whether changes in host immune mediators were altered by the treatment regime. We discovered that after recovery from cefoperazone treatment, and *H. hepaticus* infection, TNFα increased significantly while IFNγ decreased, which may be a compensatory act for the lack of IFNγ response in IL10^-/-^ mice [17]. Also, the decrease in Chi3/4 expression in cefoperazone-treated mice may be a response triggered by the decrease in microbial load after antibiotic treatment. Chitinase is highly expressed in chemically induced IBD and has a role in enhanced bacterial adhesion [67]. With lower bacterial loads, chitinase expression may also be reduced through lack of microbial stimulation. Likewise, the increases in certain mediators such as TNFα and chemokines CCL5 and CCL2 seen in the cefoperazone-treated mice, compared to untreated mice infected with *H. hepaticus,* may be due to changes in the microbial stimuli due to shifts in the microbiota after antibiotic treatment [68]. Vancomycin-treated, *H. hepaticus-*infected mice showed contrasting changes in some of the host mediators, compared to changes seen after cefoperazone administration, such as upregulation in chi3/4. Since vancomycin does not reduce total bacterial load in treated mice, unlike cefoperazone, chitinase activity may be up-regulated to facilitate bacterial adhesion for microbes present.

## Conclusion

The actual mechanism whereby the *Helicobacter* interacts with the microbiota during inflammation is yet to be determined. It is possible that the resident microbes prime the host immune system so that a response can be generated to *Helicobacter*, or interactions with other microbes are needed for *H. hepaticus* to trigger a host response. The microbiota may contribute to disease in multiple ways, making studies of potential patterns in the community important in unraveling the complexities of IBD. Understanding changes in the microbiota that are associated with IBD, can perhaps lead to novel therapies or the development of prognostic tools for early detection of this debilitating condition.

## Abbreviations

Arg: arginase; bp: base pair; CD: Crohn’s disease; CFU: colony-forming units; Chi: chitinase; CT: cycle threshold; FOX: forkhead box; GAPDH: glyceraldehyde-3-phosphate dehydrogenase; H & E: hematoxylin and eosin; IBD: inflammatory bowel disease; IFN: interferon; IL: interleukin; INDO: indoleamine-pyrrole 2,3-dioxygenase; LB: luria broth; NOS: nitric oxide synthase; OTU: operational taxonomic unit; PBS: phosphate-buffered saline; PTGS: prostaglandin-endoperoxide synthase; qPCR: quantitative polymerase chain reaction; TGF: transforming growth factor; Th: T helper; TNF: tumor necrosis factor; TSA: tryptic soy agar; TSB: tryptic soy broth; UC: ulcerative colitis

## Competing interests

The authors declare that they have no competing interests.

## Authors’ contributions

NAN and CJR contributed to experimental design; carried out animal studies, sample collection, data analyses and manuscript preparation. ILB and KAE scored histological slides. GBH helped interpret immunological changes. VBY contributed to experimental design, data interpretation, and manuscript draft. All authors read and approved the final manuscript.
